# An outbreak investigation of parechovirus-A3 in a newborn nursery

**DOI:** 10.1017/ice.2023.142

**Published:** 2024-01

**Authors:** Yuta Aizawa, Keisuke Saeki, Kazuetsu Mori, Tatsuki Ikuse, Ryohei Izumita, Akihiko Saitoh

**Affiliations:** 1 Department of Pediatrics, Niigata University Graduate School of Medical and Dental Sciences, Niigata, Japan; 2 Department of Pediatrics, Nishiwaki Municipal Hospital, Hyogo, Japan

## Abstract

**Objective:**

To investigate parechovirus-A3 (PeV-A3) transmission in a newborn nursery, after encountering 3 neonates with fever and rash.

**Design:**

An observational study.

**Setting:**

At a newborn nursery at the general hospital in Hyogo, Japan.

**Participants:**

Symptomatic neonates and their family members, and asymptomatic neonates born during the same period.

**Methods:**

PCR assays for PeV-A and genotyping were used for the investigation of PeV-A3. Preserved umbilical cords were used to identify the route of transmission.

**Results:**

PeV-A3 infection was confirmed in the three symptomatic neonates. The index case had fever and rash, and the 2 neonates treated later became symptomatic and had serum, cerebrospinal fluid, and stool specimens that were positive for PeV-A3 on PCR. The umbilical cord of the index case was positive for PeV-A3 on PCR. The family members of the index case, including the mother, were asymptomatic before delivery. The older sister and cousin of the PeV-A3–infected neonate had positive PCR results. The sequence analysis suggested 2 possible transmission routes: vertical and horizontal transmission in a newborn nursery and/or a family outside the hospital. The incubation period of PeV-A3 infection was estimated to be 1–3 days (maximum, 7 days).

**Conclusion:**

Horizontal transmission of PeV-A3 was confirmed in a newborn nursery. Vertical transmission was suggested by the detection of RNA in an umbilical cord sample from the index case. These observations indicate that PeV-A3 can be horizontally transmitted in a newborn nursery and that special caution is required to prevent healthcare-associated transmission of PeV-A3.

Parechovirus-A (PeV-A) is a nonenveloped RNA virus of the genus *Parechovirus* in the family *Picornaviridae*, which includes the enteroviruses (EVs).^1^ The spectrum of clinical presentation for PeV-A infection is wide and similar to EV infection and depends on the genotype and age of the infected hosts. Affected persons may be asymptomatic or have symptoms ranging from mild respiratory or gastrointestinal infection to severe disease.^1,2^ Among the 19 genotypes identified, parechovirus-A3 (PeV-A3) is a major cause of viral sepsis in neonates and infants aged <4 months.^1^ Although a large proportion of PeV-A3 infection is community acquired,^3^ hospital-acquired infection is rare.^4^ Only 1 healthcare-associated outbreak, in Austria, has been reported.^5^

Here, we report healthcare-associated transmission of PeV-A3 in a newborn nursery in Japan, which was identified after 3 neonates developed fever and rash. Vertical transmission of PeV-A3 was suggested by umbilical cord analysis of the index case. In addition, our observations of horizontal transmission in a newborn nursery and/or a family outside the hospital allowed us to estimate the incubation period of PeV-A3.

## Methods

### Study site

Nishiwaki Municipal Hospital is located in Hyogo Prefecture, Japan. Nishiwaki city has ∼40,000 inhabitants, and the medical area includes ∼300,000 people. The area has 2 general hospitals with pediatrics and obstetrics wards. In the newborn nursery, mothers were allowed to stay when they breastfed. The beds were usually 1 m apart, and 0.5 m apart when the newborn nursery was crowded. Healthcare workers with any symptoms such as fever, respiratory symptoms, or rash were suspended from work.

### Virological assays

Viral RNA from serum, cerebrospinal fluid (CSF), or stool was extracted with a QIAamp MinElute Virus Spin Kit (Qiagen, Valencia, CA) in accordance with the manufacturer’s instructions. When a preserved umbilical cord was available, RNA was extracted with a ZR-DuetTM DNA/RNA MiniPrep Plus Kit (Zymo Research, Irvine, CA), in accordance with the manufacturer’s instructions. Messenger RNA (mRNA) of β-actin was used as an internal control for RNA extraction from the umbilical cord. The targets of real-time reverse transcription PCR were the 5’-untranslated region for both PeV-A^6^ and EV.^7^ cDNA was synthesized with SuperScript VILO MasterMix (Invitrogen, Carlsbad, CA). Conventional PCR for mRNA of β-actin was performed with the forward primer 5’-AGAAAATCTGGCACCACACC-3’ and the reverse primer 5’-AGAGGCGTACAGGGATAGCA-3’; the band was confirmed by gel electrophoresis. The Sanger sequence was performed based on the VP3/VP1 region amplified by a nested RT-PCR assay.^8^ Genotyping was performed using BLAST analysis (https://blast.ncbi.nlm.nih.gov/Blast.cgi). The nucleotide sequences of the VP3/VP1 region have been deposited in the DNA Data Bank of the Japan/European Nucleotide Archive/GenBank databases (accession nos. LC718579–LC718583).

RNA amplification for severe acute respiratory syndrome coronavirus 2 (SARS-CoV-2) was performed using a nasopharyngeal swab and a reverse-transcriptase loop-mediated isothermal amplification assay with a Loopamp 2019-SARS-CoV-2 Detection Reagent Kit (Eiken Chemical, Tokyo, Japan).^9^ Universal screening for SARS-CoV-2 was performed for pregnant women admitted for delivery at the hospital.

This study was approved by the Ethics Committee of Nishiwaki Municipal Hospital (no. 102).

## Results

### Investigation of healthcare-associated PeV-A3 transmission

**Figure 1.**
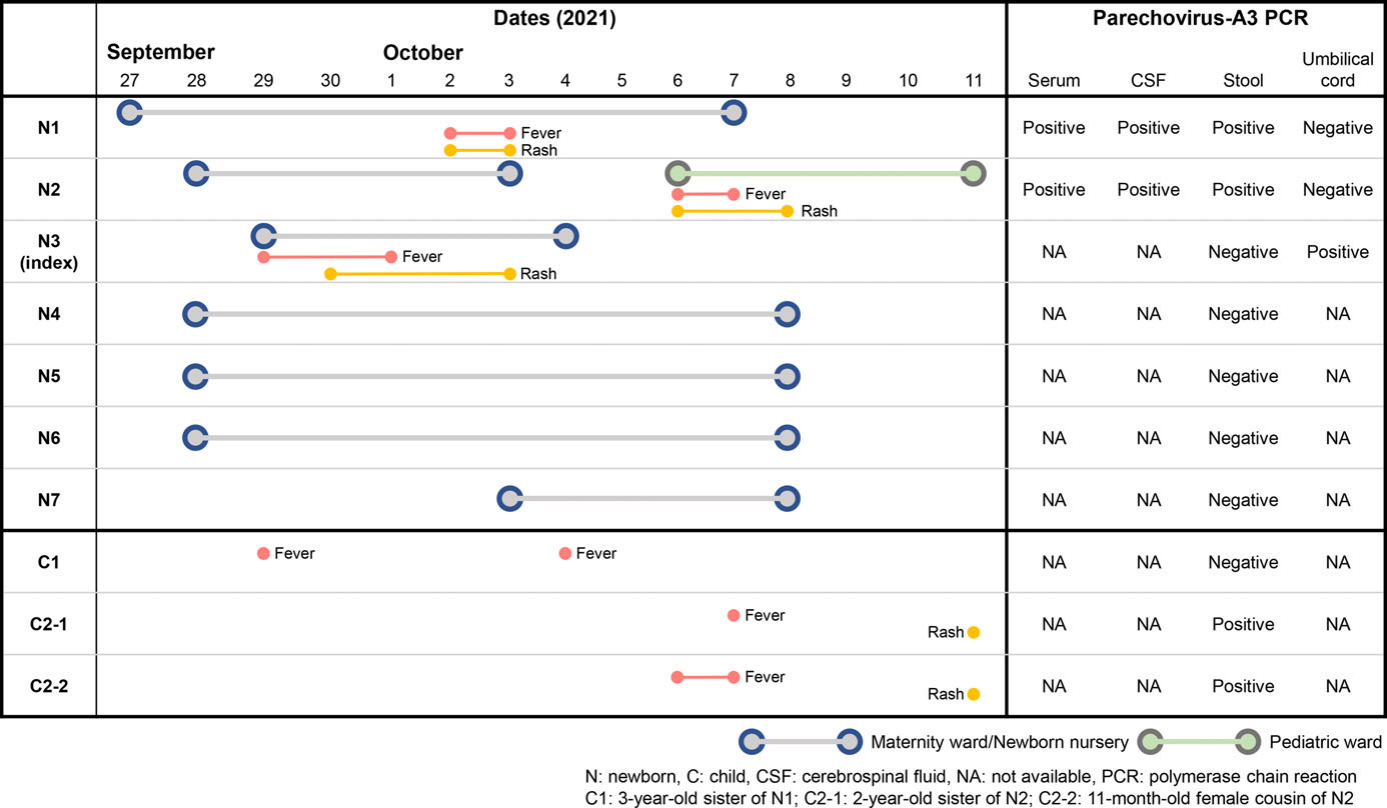
Summary of hospital stay, symptoms, and PCR results for parechovirus-A3–infected neonates in a newborn nursery and pediatric ward and their siblings and cousin at home during the study period. Grey lines indicate neonates in the maternity ward or newborn nursery, and green lines indicate neonates in the pediatric ward. Note. N, newborn; C, child; CSF, cerebrospinal fluid; NA, not available; PCR, polymerase chain reaction; C1, sister of N1 aged 3 years; C2-1, sister of N2 aged 2 years; C2-2, female cousin of N2 aged 11 months.

A full-term male neonate (gestational age, 39 weeks 1 day) with a birth weight of 2,824 g was born by vaginal delivery with Apgar scores of 8 and 8 at 1 and 5 minutes, respectively (N3, Fig. 1). No perinatal complication was noted except for the presence of green amniotic fluid at delivery. The results of nonstress antenatal cardiotocography and a cord blood examination were normal. At 5 minutes after birth, nasal flaring and retraction of the chest wall became obvious; thus, the patient was placed in an incubator for respiratory support with continuous positive airway pressure and oxygen. Body temperature was 38.5°C at 1 hour after birth. Laboratory findings were unremarkable: white blood cell count, C-reactive protein level, and immunoglobulin M level were within normal ranges; however, aspartate aminotransferase (68 IU/L), creatine phosphokinase (637 IU/L), and lactate dehydrogenase (698 IU/L) levels were slightly elevated. An erythematous maculopapular rash appeared on the trunk and extremities on day 1 and disappeared on day 4.

Other 2 full-term neonates developed fever (≤39°C) and rash after uneventful deliveries on days 3 and 7 after symptom onset of N3, respectively (N1 and N2) (Fig. 1). Both had abdominal distention and negative results for blood and CSF bacterial cultures and SARS-CoV-2 PCR. The symptoms and physical findings suggested PeV-A or EV infection. Upon request, frozen specimens of serum, CSF, and stool were sent to the laboratory of Niigata University. Positive PCR results for PeV-A3 and negative results for EV in the 3 samples from both cases (N1 and N2) led to an investigation of healthcare-associated transmission of PeV-A3 in the maternity ward and newborn nursery.

### Virological investigation

During the time the index patient (N3) and subsequent 2 febrile neonates (N1 and N2) were in the maternity ward, 4 other neonates were born at the newborn nursery (N4–N7) (Fig. 1). Approximately 1 month after the event in November 2021, stool specimens were collected from 5 patients (N3–N7; N3 is included here because the specimens were not collected at birth during hospitalization). In addition, to evaluate transmission routes of PeV-A3 from outside the hospital, stool specimens were collected ∼1 month after the event in November 2021 from older siblings and a cousin living in the same house as N1 or N2: C1 (sister of N1 aged 3 years), C2-1 (sister of N2 aged 2 years), and C2-2 (female cousin of N2 aged 11 months) (Fig. 1). The PCR results of stool specimens from C2-1 and C2-2, who were related to N2, were positive for PeV-A3.

**Figure 2.**
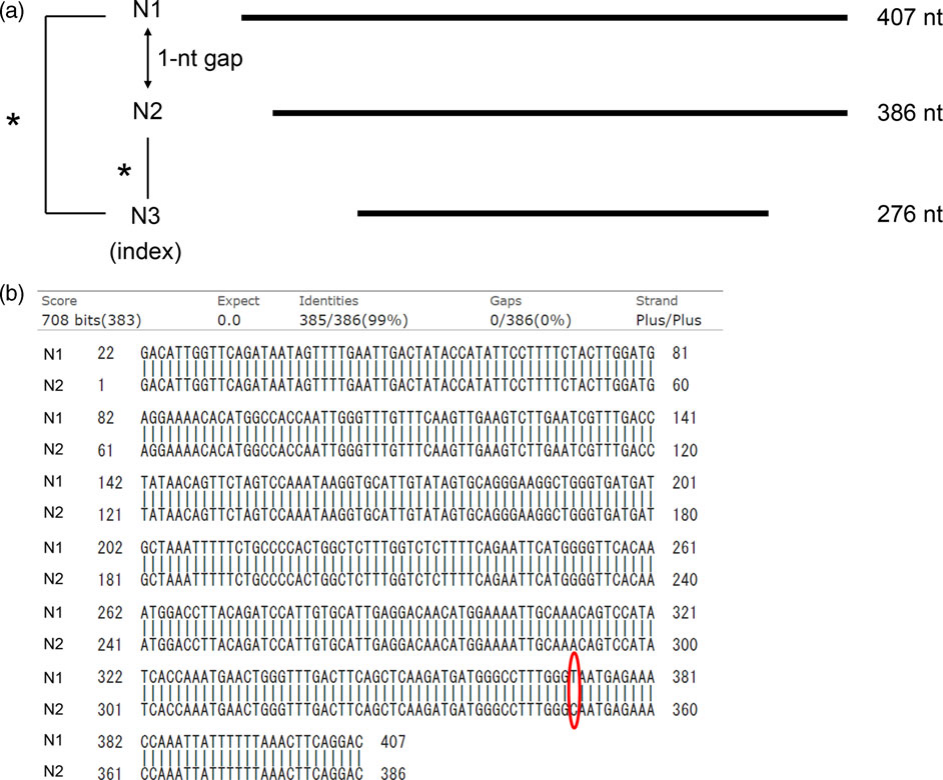
Comparison of nucleotide sequences of the VP3/VP1 region of parechovirus-A3 in parechovirus-A3–infected neonates. (a) The nucleotide sequence for N3 in an umbilical cord sample (276 nt) was identical to that for N1 in a serum sample (407 nt). The nucleotide sequences in serum samples (386 nt) were also identical for N3 and N2. (b) A 1-nucleotide difference between N1 and N2 was observed. BLAST was used for the comparison (https://blast.ncbi.nlm.nih.gov/Blast.cgi). *Indicates 100% identical.

Because the PeV-A3 infection status of C2-1 and C2-2 (ie, family members of N2) could not identify the source of PeV-A3 infection of N1 and N3, vertical transmission of PeV-A3 between N3 and the mother was suspected. Although the mothers of N1–N3 had no contact with each other before their deliveries in the maternity ward, the preserved umbilical cords of N1–N3 underwent real-time PCR for PeV-A to investigate the possibility of vertical transmission. Only the umbilical cord of the index case (N3) was positive among them, and the nucleotide sequence of VP3/VP1 in N3 was identical to that in the serum sample from N1. Although the nucleotide sequences of VP3/VP1 in serum were also identical for N3 and N2, the sequences for N1 and N2 differed by 1 nucleotide (Fig. 2a). The VP3/VP1 nucleotide sequences for N2, C2-1 (389 nt), and C2-2 (392 nt), all of whom were living in the same house, were also identical.

### Environmental investigations

The index patient (N3) received care in an incubator until October 1, 2021, and was in the open area of the newborn nursery. Patients N1–N3 shared this space in the newborn nursery through October 3, 2021. Because they were delivered by caesarean section, N4–N6 were not present in this area of the newborn nursery at this time, except during bathing, diaper change, breastfeeding, and body weight measurement in the newborn nursery. Although the bathtub was cleaned after every use, the weighing scale was not. The rules for cleaning doorknobs and handrails once or twice a day was not always followed. No healthcare workers in the newborn nursery were ill. To avoid transmission of SARS-CoV-2 during this period, hospital visits by family members were prohibited.

N2 developed a fever in the morning of October 6, 2021. The mother of N2 and C2-2 both developed fever in the afternoon of the same day, and C2-1 developed a fever the next day. The father of N2, who lived at a different location because of work, returned home on October 7, 2021, and developed fever 3 days later. Children with fever and skin rash were sporadically reported in the area near the hospital.

The mother (aged 40 years), father (aged 48 years), and older sister (aged 22 months) of N3 had been healthy and asymptomatic for 1 month before the delivery. The older sister developed hand-foot-mouth disease (HFMD) a few weeks after the delivery.

### Infection control measures

In response to the 3 consecutive neonates with fever and rash, the infection control team took action by keeping the distance between the beds >1 m, enhancing hand hygiene of staff members, and mandating proper glove- and gown-wearing and cleaning and disinfection of equipment and environmental surfaces in the newborn nursery with verbal reminders at the staff meeting.

### Estimating the incubation period of PeV-A3 infection

Our observations were used to estimate the incubation period of PeV-A3. When the source of PeV-A3 infection for N2 was considered to be different from that for N1 or N3, the incubation period of PeV-A3 infection was estimated to be 1–3 days (1 day from the end of the febrile period in N3 until the start of fever and rash in N1; 3 days from the start of the febrile period in N3 until the start of fever and rash in N1). When N3 was considered to have transmitted the disease to N1 and N2, the incubation period of PeV-A3 infection was estimated to be 1–7 days (7 days from the start of the febrile period in N3 until the start of fever and rash in N2).

## Discussion

We observed horizontal transmission of PeV-A3 infection in a newborn nursery. In addition, PCR confirmed that the umbilical cord of the index case was positive for PeV-A3, suggesting vertical transmission of PeV-A3. Notably, the mother and family members of the index case were all asymptomatic, which made it difficult to predict the source of infection.

Vertical transmission of PeV-A3 was suggested by our observations. Although 3 previous cases of suspected vertical transmission of PeV-A3 were reported (Table 1),^10–12^ samples collected at birth, such as cord blood and umbilical cord, were unavailable. Thus, to our knowledge, this is the first case in which a PeV-A was detected in a sample collected at birth. Japanese maternity hospitals customarily provide umbilical cord to parents as a birth memento. This tissue is very important as evidence of vertical transmission of viruses because it directly connects mother and fetus. Preserved umbilical cord has been used to confirm vertical transmission of congenital cytomegalovirus,^13^ rubella,^14^ and neonatal enteroviral infection.^15^ The viral genome can be detected long after preservation of the tissue, which can thus be used retrospectively for viral diagnosis.

**Table 1.** Summary of Case Reports on Vertical Transmission of Parechovirus-A3

Case No.	GA; Birthweight	Fetal Distress^a^	Clinical Diagnosis	Outcome	Mother	Maternal Positive PCR	Neonatal Positive PCR	Other Sick Contact	Reference
1	32 4/7 weeks; 2,347 g	Reduced movement; absence of baseline heart rate variability	Meningoencephalitis	Severe neurodevelopmental delay at 2.8 years	Mild gastroenteritis 1–2 weeks before	NA	Stool (day 3), CSF (day 5)	NA	Salavati et al^10^
2	38 3/7 weeks 4,198 g	Reactive tachycardia while mother was febrile	Meningoencephalitis	Most likely to develop cerebral palsy at 4 months	Fever, malaise, myalgia, and rash 1 week before	Plasma and swabs (nasal, amnion, and chorion)	Plasma and swabs (nasal and rectal) (day 1), CSF (day 3)	Sibling aged 2 years; grandmother^b^	Hilbig et al^11^
3	36 4/7 weeks 3,010 g	Reduced movement; tachycardia	Encephalitis	Cerebral palsy at 4 months	NA	NA	Swabs (throat and rectal) (day 1), CSF (day 3)	Sibling aged 2 years (fever and rash 10 days prior)	Mei et al^12^
4	39 1/7 weeks 2,824 g	Meconium-stained amniotic fluid	Sepsis	Normal development at 10 months	Asymptomatic	NA	Umbilical cord	None	Present case

Note. GA, gestational age; CSF, cerebrospinal fluid; PCR, polymerase chain reaction; NA, not available.

a Fetal heart rate was assessed by cardiotocography.

b Symptoms were not described for 2 persons.

Because PeV-A3 is transmitted by the fecal–oral or respiratory routes,^1^ contact and droplet precautions must be added to standard precautions.^16^ Even asymptomatic individuals shed PeV-A3 that can be a source of infection,^17^ and adults are more likely than children to be asymptomatic.^18^ Hand hygiene, cleaning and disinfection of equipment and environmental surfaces, and cohort placement of infected neonates are important for controlling hospital nursery outbreaks. Contact precaution is adequate while infected neonates are symptomatic.^19^ Frequent hand hygiene is essential: Although an alcohol-based hand sanitizer containing at least 60% alcohol is adequate, use of soap and water is preferred for a nonenveloped virus such as PeV-A3 when care includes diapering or toileting and eating or feeding.^16^ Our present observations show that early suspicion and diagnosis of PeV-A3 infection in neonates in a newborn nursery enabled adequate infection control measures, which limited the PeV-A3 outbreak to a small number of patients. However, prevention of transmission of PeV-A3 to a newborn nursery was challenging because the source of infection was an asymptomatic pregnant woman admitted for delivery.

Horizontal transmission in an enclosed space enabled us to estimate the incubation period of PeV-A3. To our knowledge, only 1 previous study reported an estimate of the PeV-A3 incubation period, which was derived from healthcare-associated transmission in a maternity ward.^5^ The authors assumed, without direct evidence, that the source of PeV-A3 infection was asymptomatic adults or siblings and that the incubation was 1–12 days, which is consistent with our observation of an incubation period no longer than 7 days.

During the 2014 PeV-A3 epidemic in Japan, the youngest patient was aged 4 days,^3^ which is also consistent with our estimated incubation period. However, because preserved umbilical cord was unavailable, it was unclear whether this was a case of vertical or horizontal transmission. The 1-nucleotide difference in longer sequences from N1 and N2 and the fact that the family members of N2 developed symptoms outside the hospital at the same time as N2 suggest that the source of PeV-A3 infection differed for N1 and N2, that is, horizontal transmission from N3 to N1 in the newborn nursery and horizontal transmission from community-dwelling persons to N2. In the area near the hospital, pediatricians reported children with fever and skin rash during the period from September through October 2021. In fact, pediatric sentinel surveillance in the prefecture showed herpangina from June 2021^20^ and HFMD from July 2021,^21^ although passive pathogen surveillance did not detect PeV-A in patients with herpangina or HFMD.^22^ National surveillance of data aggregated from local health laboratories detected 16 PeV-A3 strains in 2021, one of which was from a patient with HFMD.^23^ In 2021, during the SARS-CoV-2 pandemic, the number of PeV-A3 infections decreased substantially.^24^

The limitations of this study should be acknowledged. No sample was available from the index case (N3) during acute illness or the asymptomatic mother at delivery. Such samples could provide information that would confirm vertical transmission of PeV-A3. Similarly, samples from other family members of the index case, including the older sister with HFMD a few weeks after delivery and the asymptomatic father, would have helped identify the source of PeV-A3 in the family.

In conclusion, we confirmed horizontal transmission of PeV-A3 in a newborn nursery. Vertical transmission of PeV-A3 infection was suggested by a positive PCR result for an umbilical cord sample. Early suspicion and diagnosis of PeV-A3 infection were crucial in preventing healthcare-associated transmission of PeV-A3 in a newborn nursery.
